# Systematic analysis of prognostic significance, functional enrichment and immune implication of STK10 in acute myeloid leukemia

**DOI:** 10.1186/s12920-022-01251-7

**Published:** 2022-05-01

**Authors:** Lei Bi, Shuangshuang Jia, Wuyue Hu, Xiaoli Su, Xiequn Chen, Hailong Tang

**Affiliations:** 1 Department of Hematology, Xijing Hospital, Air Force Military Medical University, Xian, 710032 Shaanxi People’s Republic of China; 2 Institute of Hematology, Northwest University, Xian, 710069 Shaanxi People’s Republic of China; 3 Department of Hematology, Affiliated Hospital, Northwest University, Xian, 710082 Shaanxi People’s Republic of China

**Keywords:** STK10, AML, Prognosis, Functional analysis, Immune infiltration

## Abstract

**Background:**

Despite deeper understanding of the genetic landscape of acute myeloid leukemia (AML), the improvement of survival is still a great challenge. *STK10* is overexpressed in several cancers with functions varying according to cancer types. But the functions of *STK10* in AML has never been reported.

**Methods:**

We analyzed the expression, prognosis and potential functions of *STK10* utilizing public web servers. Metascape and the String database were used for functional and protein–protein interaction analyses.

**Results:**

We found *STK10* was enriched in blood & immune cells and overexpressed in AML. High *STK10* expression was associated with poor overall survival, which was also identified in the subgroups of patients ≤ 60 years old and patients with non-high-risk cytogenetics. We demonstrated genes associated with *STK10* were enriched in blood, spleen and bone marrow, influencing the immune function and biological process of AML. *ITGB2* and *ITGAM* might directly interact with *STK10* and were associated with poor prognosis. Besides, *STK10* was associated with the infiltration of immune cells and immune checkpoints, like *HLA-E*, *CD274* and *GAL-9*.

**Conclusions:**

The present study was the original description of *STK10* in AML and set the stage for developing *STK10* as a new prognostic marker or therapeutic target for AML.

**Supplementary Information:**

The online version contains supplementary material available at 10.1186/s12920-022-01251-7.

## Background

Acute myeloid leukemia (AML), characterized by infiltration of the clonal, abnormally or poorly differentiated and highly proliferative cells in bone marrow (BM) or blood, is the second commonly known leukemia in adults. Despite progress in the genetic landscape and the followed transformation into promising therapies, the improvement of prognosis is still a great challenge for clinicians owing to the enormous molecular diversity [1]. Therefore, there is still an urgent need to identify new prognostic factors or therapeutic targets for AML.

The *serine/threonine kinase 10* (*STK10*), also called *lymphocyte-oriented kinase* (*LOK*) and *PRO2729*, is located on nucleoplasm and plasma membrane [2]. The production of *STK10* is mainly synthesized in lymphocytes and shows homology to the *STE20* family members, notably involved in the mitogen-activated protein kinase (MAPK) cascades [3]. It has been demonstrated that the *STK10* might act as a tumor suppressor in the aggressive lymphoma [4] but implicated an pro-tumor action in ewing sarcoma [5], indicating that *STK10* might play diverse functions according to different tumor types.

A vital function of *STK10* is to phosphorylate the *polo-like kinase 1* (*PLK1*) [3], which has been found to be involved in the regulation of the cell cycle, especially in the G2/M transition and during mitosis [6–9]. *PLK1* is overexpressed in several solid tumors and leads to poor prognosis, as well as in AML [7, 9, 10]. *STK10* also functions as an ezrin-radixin-moesin (ERM) kinase, activating the ERM family of proteins which are involved in the development and metastasis of various types of cancers, like prostate cancer, breast cancer and rhabdomyosarcoma, etc. [3, 11–13]. The knockout or caspase cleavages of *STK10* result in dephosphorylation of ERM, further inhibiting cell migration [3, 11]. According to a recent study, *ezrin* (*EZR*), a member of ERM, has been identified as a marker of poor prognosis in AML. *EZR* inhibitors successfully inhibited the viability and autonomous clonal growth in AML cells [14]. These observations may suggest an indication of the regulating function of *STK10* in AML. However, the potential functions of *STK10* in AML have not been explored so far.

In the present study, we uncovered the characteristic of *STK10* expression in AML, as well as the prognostic values of *STK10*. Then we investigated the functions of *STK10*-associated genes and the interaction between *STK10* and the STK10-related proteins. Finally, the relationship between *STK10* expression and immune cell infiltration in AML was also explored. Our work has made some contributions to our understanding of the biological functions of *STK10* in AML.

## Methods

### Human Protein Atlas (HPA) analysis

HPA (https://www.proteinatlas.org) is open access for users. It provides a huge atlas by various omics technologies, including antibody-based imaging, mass spectrometry-based proteomics, transcriptomics and systems biology [2]. We acquired STK10 expression in different cell types by searching the term “*STK10*” in the field of “Cell Type category (RNA)”. The result provides a summary of single-cell RNA, denoted by normalized expression (NX) of transcripts per million (TPM), from all normal single cell types.

### Gene Expression Profiling Interactive Analysis (GEPIA) analysis

GEPIA (https://www.gepia.cancer-pku.cn) is developed based on the RNA-seq data of 9,736 tumors and 8,587 normal samples from the Cancer Genome Atlas (TCGA) and the Genotype-Tissue Expression project (GTEx) [15]. The general module on the homepage of the website provides the summary information and gene expression profile. The bar plot was adopted to present *STK10* expression across all tumor samples and paired normal tissues, in which the ordinate offers TPM values of samples.

### GEO, TCGA and GETx data download and process

To compare the expression of *STK10* between tumor and normal tissues, we acquired the TPM format of RNA-seq data in TCGA and GTEx database that uniformly processed by Toil’s method [16] from UCSC Xena (https://xenabrowser.net/datapages/). Then we download the latest RNA-seq and clinical information from the TCGA database (https://portal.gdc.cancer.gov/). The data from the TCGA database was transformed from HTSeq-fragments per kilobase per million (FPKM) to TPM for the following analysis.

Besides, we acquired expression data of GSE9476 [17] from the Gene Expression Omnibus (GEO) repository (https://www.ncbi.nlm.nih.gov/gds/) to verify the results we concluded. GSE9476 provides expression profiling by array, collected from leukemic blasts of 26 AML patients and CD34+ selected cells of 8 BM of health donors. The log2 expression value obtained from gcRMA analyses was processed in our analysis.

### Metascape analysis

Metascape (http://metascape.org) is a practical tool to infer enriched biological pathways. By submitting overlapping genes in the multiple gene list module, functional enrichments in Gene Ontology (GO), Kyoto Encyclopedia of Genes and Genomes (KEGG) pathways were analyzed automatically [18]. Metascape database also provides enrichment analysis with Pattern Gene Database (PaGenBase) [19], providing further views in cells, tissues or organs the genes enriched in, and Transcriptional Regulatory Relationship Unraveled by Sentence-based Text mining (TRRUST) [20], offering key regulatory factors of the gene list.

### String analysis and process

The String database version 11.0b (https://string-db.org/) could provide known and predicted protein–protein interactions (PPI) covering 24,584,628 proteins from 5090 organisms [21]. We employed the multiple proteins module to identify existing proteins by submitting the genes associated with *STK10*, and further constructed the PPI network. Through the Cytoscape software (v3.8.0) [22], we disposed of the string interaction file, produced by the String database automatically, for visualization of the network.

### Immune infiltration

ssGSEA algorithm was used to assessment the level of immune infiltration [23]. The method uses a gene set which provides specific makers of immune cells to calculate the enrichment score of immune cells. After getting the level of immune infiltration in each sample, the Spearman’s test was used for exploring the correlation of *STK10* with immune infiltration.

### Statistical analysis

Analyses of samples were processed using R (version 3.6.3). We classified the data into two groups according to the median value of *STK10* expression. Categorical variables, like sex, age, cytogenetic risk and French-American-British (FAB) classifications, were tested by Pearson’s chi-square and Fisher’s exact tests. Continuous variables mainly included White blood cell (WBC) count, BM blasts, peripheral blood (PB blasts) and gene expression. If the continuous variable does not obey normal distribution, examined by the Shapiro–Wilk test, the Mann–Whitney U test was used for the comparison, otherwise the Student-t test was adopted. Gene expression between *STK10*^high^ and *STK10*^low^ groups with the absolute value of log2 fold change (log_2_FC) > 1 and *P.adj* < 0.5 was considered as differentially expressed. The Spearman’s test was implemented to probe the correlation between *STK10* and related genes, as well as immune cell infiltration level. Overall survival (OS) was compared using the Log-rank testing. *P* value < 0.05 was considered as significant (ns: *P* > 0.05, *: *P* < 0.05, **: *P* ≤ 0.01, ***: *P* ≤ 0.001).

Univariate and multivariate survival analyses was carried out using Cox regression models to test the function of STK10 in predicting patients’ survival. The multivariate survival analysis would be performed based on factors with statistical significance (*P* < 0.05) in the univariate analysis.

R packages adopted in this research included ggpolt2 package (v_3.3.3) and survminer package (v_0.4.9) for visualization, pROC package (v_1.17.0.1) for drawing receiver operating characteristic (ROC) curve, survival package (v_3.2-10) for analyzing OS, DEseq2 package (v_1.26.0) for figuring out the differential genes with STK10, and stat package (v_3.6.3) for correlation analysis with *STK10*. The correlation between *STK10* and immune infiltration was analyzed by the GSVA package (v_1.34.0).

## Results

### *STK10* overexpression in AML

**Fig. 1:**
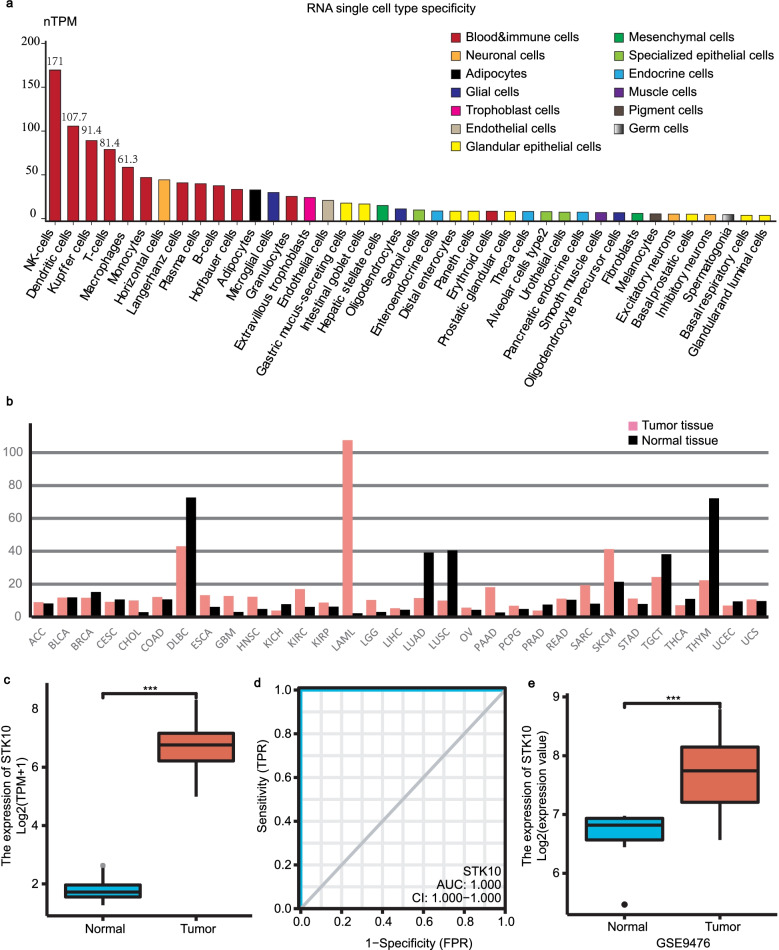
*STK10* expression in different cell types and tumors. **a** *STK10* expression in different cell types, analyzed by HPA. **b** *STK10* expression across various tumors and normal tissues, analyzed by GEPIA. **c** The expression of *STK10* between normal tissues and AML based on data from TCGA and GETx. **d** ROC curve based on *STK10* expression in distinguishing normal tissues and AML. **e** *STK10* expression on AML and health donors in GSE9476

To elucidate the cell type specificity of *STK10* expression, we first analyzed *STK10* expression based on the RNA-seq data from the HPA database. We listed the top 40 cell types based on nTPM (normalized transcripts per million) value of *STK10* and numbered the top five with the value of nTPM*.* The levels of *STK10* mRNA expression were higher in blood &immune cells, like NK cells, Dendritic cells, kupffer cells, T-cells and macrophages (Fig. 1a). Subsequently, we tested the expression of *STK10* in different types of cancers by comparing the RNA-seq data of TCGA and GTEx projects in the GEPIA database. The results revealed that the level of *STK10* expression was up-regulated significantly in AML (Fig. 1b). Figure 1c uncovered the difference of *STK10* expression between 173 AML and 70 normal BM samples individually (*P* < 0.001). The ROC curve was also adopted to examine the capacity of *STK10* to distinguish AML from normal samples. As presented in Fig. 1d, the area under the curve (AUC) was 1.00 (*P* < 0.001), suggesting the expression of *STK10* could be an ideal marker to distinguish AML from normal tissues. Then we verified the expression of *STK10* in GSE9476, another dataset from GEO. And the differential expression of *STK10* was also observed (Fig. 1e, *P* < 0.001).

### Relationships between *STK10* and clinicopathological characteristics of patients with AML

We stratified 151 patients into two groups based on the median value of the *STK10* expression (median value, 77.64). Characteristics between the two groups were summarized in Table 1. 75 patients were classified into the *STK10*^low^ group, and 76 patients were in the *STK10*^high^ group. Significant differences were observed in age grouped by 60 years old (*P* = 0.025) and cytogenetic risk stratification (*P* < 0.001) between *STK10*^low^ and *STK10*^high^ groups. No significant differences existed in gender, WBC count, BM blast, PB blasts and FAB classification.

**Table 1:** Correlations between *STK10* expression and clinicopathological features in AML from TCGA cohort

Characteristics	*STK10* expression		*P* value
	Low (n = 75)	High (n = 76)	
Sex, male/female	43/32	40/36	0.677
Age, n (%)			0.025
≤ 60	51 (33.8%)	37 (24.5%)	
> 60	24 (15.9%)	39 (25.8%)	
WBC (10^9^/L), meidan (Q1–Q3)	14 (4, 41.5)	26 (6, 75.5)	0.075
BM blasts(%), meidan (Q1–Q3)	40 (5, 64)	36 (11, 64.75)	0.518
PB blasts(%), meidan (Q1–Q3)	72 (49.5, 85.5)	71 (50.75, 84.25)	0.863
Cytogenetic risk, n (%)			< 0.001
Favorable	27 (18.1%)	4 (2.7%)	
Intermediate	31 (20.8%)	51 (34.2%)	
Poor	16 (10.7%)	20 (13.4%)	
FAB classifications, n (%)			0.183
M0	7 (4.7%)	8 (5.3%)	
M1	12 (8%)	23 (15.3%)	
M2	20 (13.3%)	18 (12%)	
M3	12 (8%)	3 (2%)	
M4	14 (9.3%)	15 (10%)	
M5	7 (4.7%)	8 (5.3%)	
M6	1 (0.7%)	1 (0.7%)	
M7	1 (0.7%)	0 (0%)	

*n* number of patients, *WBC* white blood cell, *BM* bone marrow, *PB* peripheral blood, *FAB* French–American–British, *Q1* lower quartile, *Q3* upper quartile

**Fig. 2:**
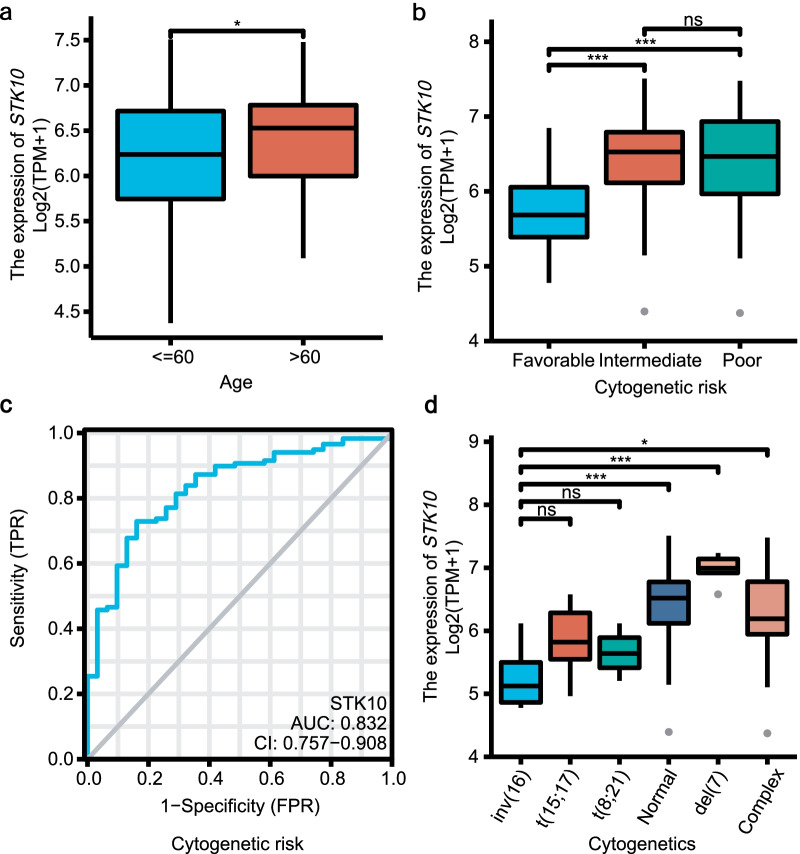
Correlation between *STK10* expression and clinical characteristics, based on data from TCGA. **a** *STK10* expression in AML with different ages. **b** *STK10* expression in AML with different cytogenetics risk stratifications. **c** ROC curve based on *STK10* expression in predicting the favorable cytogenetic risk. **d** *STK10* expression in AML with specific cytogenetics

We next tested the expression of *STK10* in different groups of age and cytogenetic risk stratification. In the patients > 60 years old, the expression of *STK10* was higher when compared to the patients ≤ 60 years old (*P* = 0.034, Fig. 2a). *STK10* was overexpressed in intermediate- (*P* < 0.001) and poor- (*P* < 0.001) risk groups compared with the favorable group (Fig. 2b). And there was no difference between intermediate- and poor- risk groups. Based on the ROC curve, we found that low expression of *STK10* could serve as a diagnostic factor in predicting the favorable cytogenetic risk with medium accuracy (AUC = 0.832, CI 0.757–0.908, Fig. 2c. We further evaluated the *STK10* expression in specific cytogenetics (Fig. 2d). *STK10* expression was the lowest in the inv(16) group, showing no difference with that in the t(15;17) and t(8;21) groups, compared with the intermediate- or poor- risk groups. In addition, the expression of *STK10* indicated no statistical significance between the intermediate- and poor- risk groups.

### High *STK10* expression is associated with a poor prognosis of AML

**Fig. 3:**
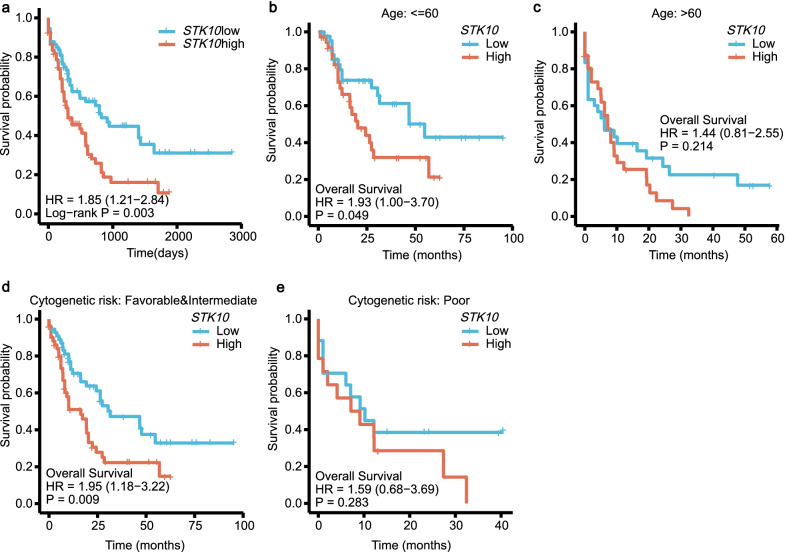
Prognostic values of *STK10* in AML patients, based on TCGA datasets. **a** Survival curves of OS between the *STK10*^low^ and *STK10*^high^ groups. Prognostic values of *STK10* had been shown in the subgroup of the patients ≤ 60 years (**b**), the patients > 60 years (**c**), the patients with favorable&intermediate cytogenetic risks (**d**) and the patients with poor cytogenetic risks (**e**)

The association between *STK10* expression and OS of patients with AML was evaluated by Kaplan–Meier (K-M) analysis, which indicated that low expression of *STK10* was correlated with favorable OS in AML (HR 1.85, CI 1.21–2.84, *P* = 0.003, Fig. 3a). We further explored the relationship between *STK10* expression and OS of the specific subgroups. As shown in Fig. 3b, c, among the patients ≤ 60 years old, the patients with *STK10*^low^ expression had higher OS when compared with those with *STK10*^high^ expression (*P* = 0.049). However, the OS benefit of *STK10*^low^ expression could not be seen among the patients > 60 years old (*P* = 0.214). Similarly, the significant difference in OS between the *STK10*^low^ and *STK10*^high^ expression could only be found in the favorable & intermediate risk group (*P* = 0.009, Fig. 3d, e).

We performed univariate and multivariate survival analyses based on patients’ clinical characteristics and the expression of STK10 by cox regression analysis. As presented in Additional file 1, the *P* values of age, cytogenetic risk and STK10 in the univariate analysis show statistical significances. In further multivariate analysis, the HR (95% CI) between *STK10*^low^ and *STK10*^high^ groups is 1.562 (1.001–2.438) with statistical significance (*P* = 0.050). This result indicates the expression of STK10 could predict patients’ survival as an independent prognostic factor.

### ***STK10*** associated gene analysis between ***STK10***^high^ and ***STK10***^low^ groups in AML

**Fig. 4:**
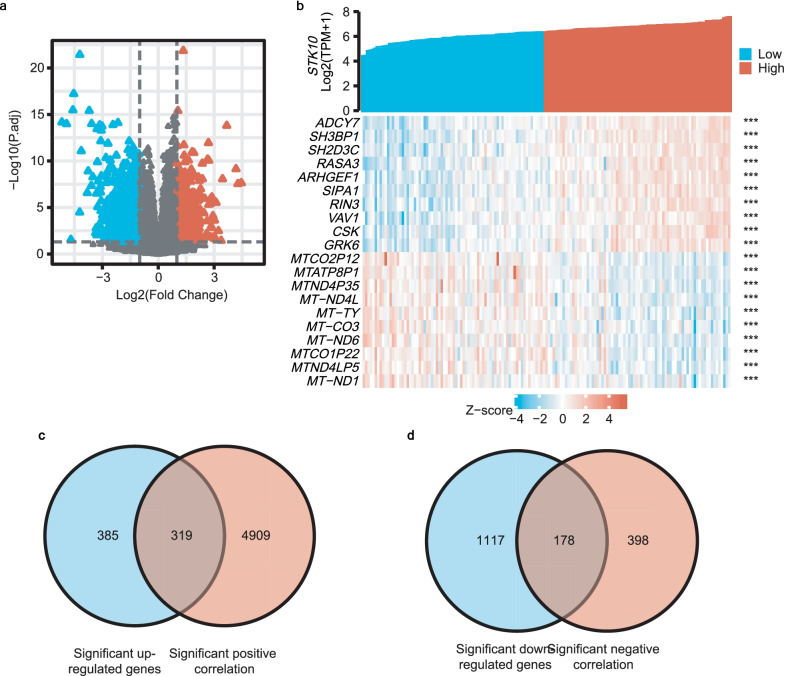
Genes associated with *STK10* expression. **a** Volcano plot of different gene expression profiles between *STK10*^high^ and *STK10*^low^ groups. **b** Top ten co-expression genes positively and negatively associated with *STK10* based on Spearman test’s value. **c** Overlapping genes that were significantly up-regulated and positively correlated with *STK10*. **d** Overlapping genes that were significantly down-regulated and negatively correlated with *STK10*

In order to further evaluate the functional role of *STK10* in AML, we first analyzed the expression of differential genes between *STK10*^low^ and *STK10*^high^ groups based on the TCGA database. A total of 1999 genes had a significant difference between *STK10*^high^ and *STK10*^low^ groups (*P*.adj < 0.05, |log_2_FC|> 1, see Additional file 2), including 704 up-regulated genes (red triangle) and 1295 down-regulated genes (blue triangle) as shown in the volcano plot (Fig. 4a).

Then the correlation of genes in conjunction with *STK10* expression was identified by the Spearman’s correlation test. A total of 5804 gens were found correlated with *STK10* expression (*P* < 0.05, and |cor|≥ 0.3, Additional file 3). We listed the top ten of 5228 positively correlative genes and the top ten of negatively 576 correlative genes in Fig. 4b. Among the positively correlative genes, 319 genes were significantly up-regulated (Fig. 4c). And 178 genes were significantly down-regulated in the negatively correlative genes (Fig. 4d). The overlapping genes were processed into biological function analysis and PPI analysis subsequently.

### Biological function of *STK10*-associated overlapping genes

**Fig. 5:**
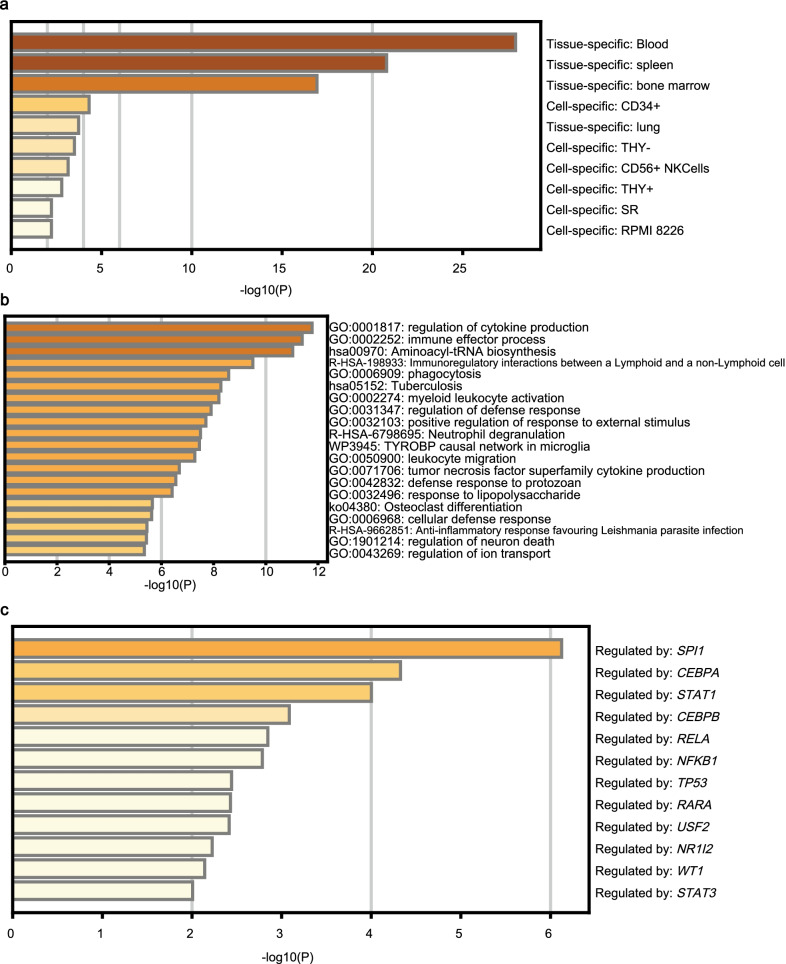
Functional analysis of the overlapping genes in AML. **a** Enrichment of the overlapping genes in tissues and cells based on PaGenBase. **b** Analysis of GO and KEGG pathways associated with *STK10*. **c** The relevant regulatory genes of the overlapping genes based on the TRRUST

We then investigated the biological function of the 497 overlapping genes in the Metascape database. As listed in Fig. 5a, the summary of enrichment analysis in PaGenBase revealed the overlapping genes were mainly enriched in blood, spleen and BM, indicating their potential function in the hematological system. Through the GO/KEGG pathway annotations, we found that these genes were associated with several biological processes of leukemia, like regulation of cytokine production, phagocytosis, myeloid leukocyte activation, leukocyte migration and tumor necrosis factor superfamily cytokine production (Fig. 5b). Besides, three clusters belonged to the immune system, including the immune effector process, regulation of defense response, and immunoregulatory interactions between a Lymphoid and a non-Lymphoid cell. Besides, relevant regulatory genes of the overlapping genes provided by TRRUST were presented in Fig. 5c, some of which have been identified as crucial factors in leukemogenesis, such as *SPI1*, *CEBPA*, *STAT1*, *TP53*, *RARA* and *WT1* [24–29].

### PPI identifies the STK10-related proteins

**Fig. 6:**
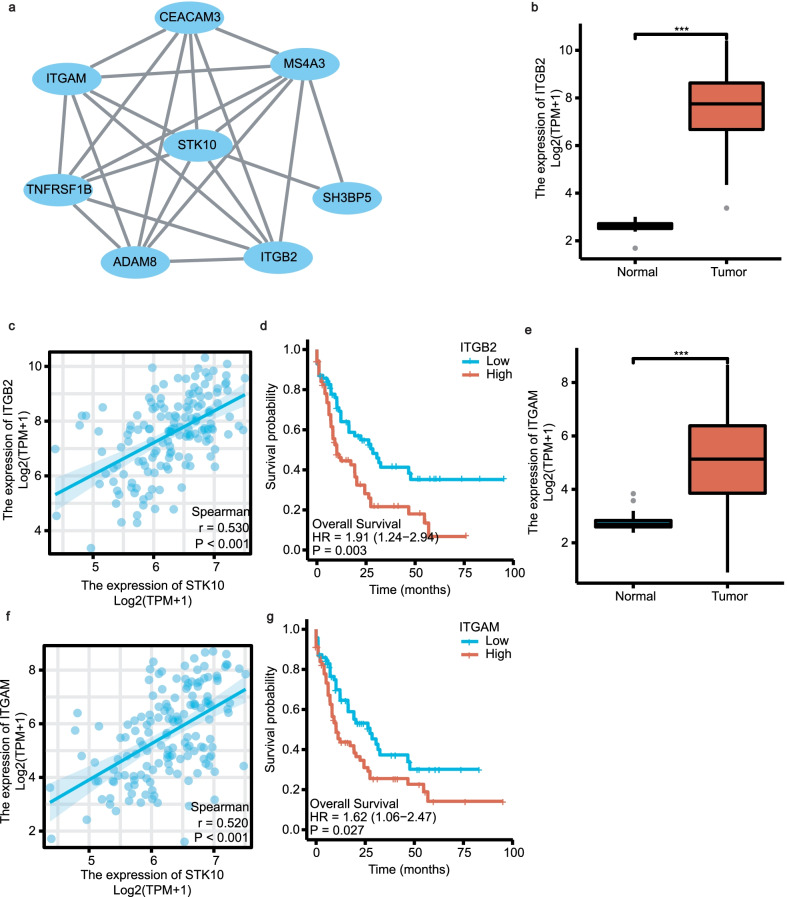
PPI network of the overlapping genes. **a** Genes from (Additional file 4) interacted with STK10 directly. **b**, **e** The expression of *ITGB2* and *ITGAM* between normal tissues and AML. **c**, **f** The co-expression of *ITGB2* and *ITGAM* with *STK10*. **d**, **g** The prognostic value of *ITGB2* and *ITGAM* in AML

The PPI analysis was conducted by inputting the 497 overlapping genes into the String database. As a result, 172 nodes and 631 edges were obtained (Additional file 4). The nodes colored yellow were the proteins that directly interacted with STK10. We showed the proteins at length in Fig. 6a, including 8 nodes and 23 edges. Among them, *ITGB2* (also called LFA-1) and *ITGAM* were overexpressed in AML (Fig. 6b, e) and positively correlated with *STK10* (*P* < 0.001, Fig. 6c, f). According to the K-M plot, *ITGB2* and *ITGAM* were also related to poor OS in AML (*P* = 0.003 and *P* = 0.027 respectively, Fig. 6d, g). The expression of *ITGB2* and *ITGAM* in the dataset GSE9476 was also higher in tumor samples compared with donors (Additional file 5a, c). Both *ITGB2* and *ITGAM* was positively correlated with *STK10* expression in GSE9476 (Additional file 5b, d).

In Fig. 6a, seven genes were found to be associated with *STK10* directly in the protein–protein interactions analysis, including *CEACAM3*, *ITGAM*, *TNFRSF1B*, *ADAM8*, *ITGB2*, *SH3BP5* and *MS4A3*. Mann–Whitney U test was adopted to compare the expression of these genes between normal tissues and tumor samples from AML. And five genes (*CEACAM3*, *ITGAM*, *ADAM8*, *ITGB2* and *SH3BP5*) were selected to enter the univariate and multivariate survival analyses with *STK10* to prove that whether high STK10 expression is an independent prognostic indicator in AML.As shown in Additional file 6, high level expression of *ITGAM*, *ITGB2* and *STK10* own statistical significances (*P* < 0.05) in the univariate analysis. In the further multivariate analysis, the HR (95% CI) between the high expression level and low expression level of *STK10* is 1.610 (1.015–2.554) with statistical significance (*P* = 0.043), indicating *STK10* could act as a prognostic indicator for patients with AML at the genetic level.

### STK10 expression and immune cell infiltration in AML

**Fig. 7:**
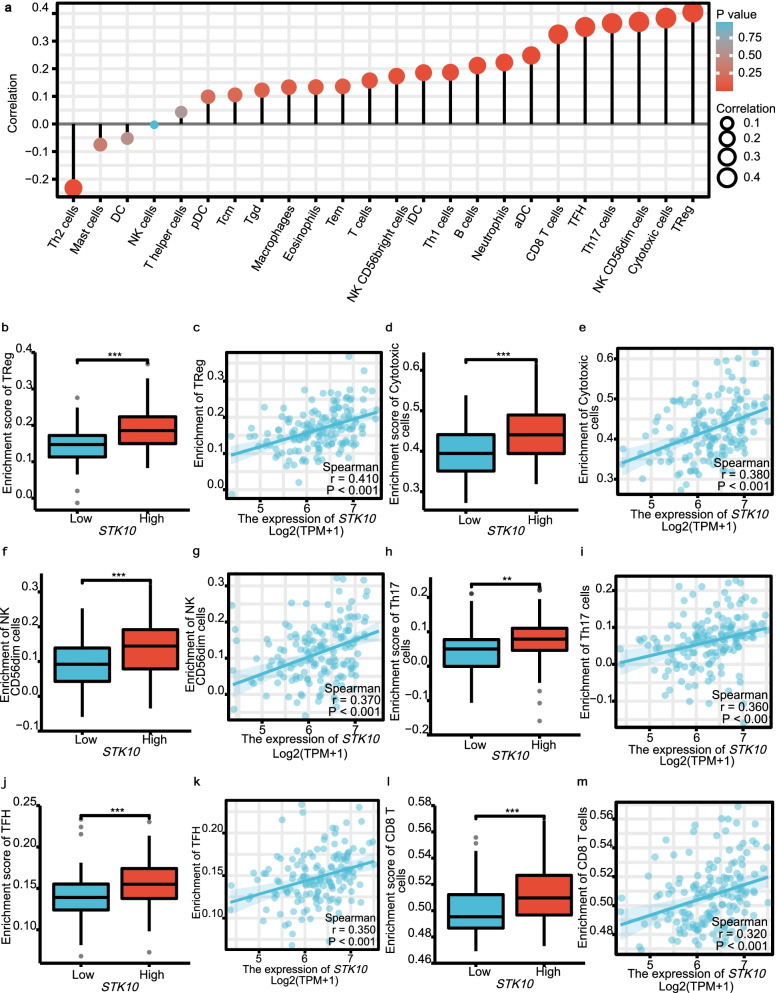
The relationship between *STK10* and the immune cell infiltration. **a** Forrest plot showing the connection between *STK10* and the immune cell infiltration levels. **b**, **d**, **f**, **h**, **j**, **l** The levels of various immune cells (|cor|≥ 0.3, *p* < 0.05) in *STK10*^low^ and *STK10*^high^ groups. **c**, **e**, **g**, **i**, **k**, **m** The correlation of *STK10* expression with the specific immune cell infiltration levels. Cytotoxic cells: including CD8 T cells, Tgd, and NK cells

The results above suggested that *STK10* associated genes were involved in immunologic function (Fig. 5b). Thus, we further explored the relationship between *STK10* and immune cell infiltration in AML. As shown in Fig. 7a, *STK10* was significantly positively correlated with several immune cells, like regulatory T cells (TReg), CD8 T cells, T follicular helper (TFH), Th17 cells, NK CD56^dim^ cells and cytotoxic cells (|cor|≥ 0.3, *P* < 0.05). Grouped by the median value of *STK10* expression, the infiltration levels of all these immune cells were significantly higher in the *STK10*^high^ group (Fig. 7b, d, f, h, j, l). The correlation of *STK10* expression with immune cell infiltration was also evaluated (Fig. 7c, e, g, i, k, m), revealing the positive association with *STK10*.

**Fig. 8:**
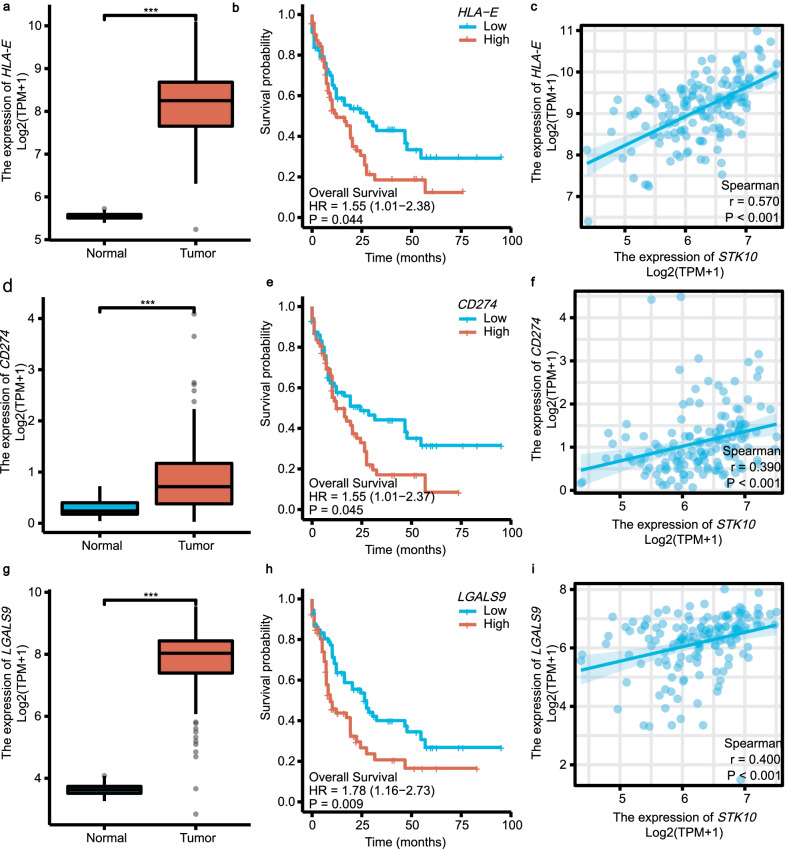
The roles of specific immune checkpoints in AML. **a**, **d**, **g** The expression of *HLA-E*, *CD274* and *LGALS9* between normal tissue and AML. **b**, **e**, **h** The prognostic value of *HLA-E*, *CD274* and *LGALS9* in AML. **c**, **f**, **i** The co-expression of *HLA-E*, *CD274*, *LGALS9* with *STK10*

We know that NK cells and cytotoxic T lymphocytes (CTLs) exert the antitumor effect through the combination of the receptors on the surface of the effector cells and the corresponding ligands on the surface of the target cells [30–32]. Therefore, we explored the association between *STK10* expression and these ligands on the surface of AML cells, such as HLA-A, HLA-B, HLA-C, HLA-E, PD-L1(CD274), PD-L2 and GAL-9(produced by *LGALS9*), etc. Among them, *HLA-E*, *CD274* and *LGALS9* were overexpressed on tumor cells (Fig. 8a, d, g) and predicted poor OS in AML (Fig. 8b, e, h). Moreover, Spearman’s correlation test revealed the positive correlation between these ligands on tumor cells with *STK10* (Fig. 8c, f, i). We also explored the expression of *HLA-E* and *LGALS9* in GSE9476. The results were consistent with our findings above that *HLA-E* was overexpressed on tumor cells and positively correlated with *STK10* expression (Additional file 5e, f). Although the expression of *LGALS9* on tumor samples was higher as well, the spearman’s correlation test showed there was no statistical correlation between *STK10* and *LGALS9* (Additional file 5g, h).

## Discussion

AML is a highly heterogeneous disease with different outcomes even among patients with the same known genetic background, indicating more accurate prognostic and therapeutic targets need to be developed. The possible mechanisms of the poor prognosis include the existence of leukemia stem cells, the interaction with BM microenvironment, immune evasion or clonal evolution leading to primary or acquired chemoresistance. The overexpression and potential pathogenetic significance of *STK10* in multiple cancers have been observed [5, 11, 33]. However, the relationship between *STK10* and AML has not been examined to date and needs to be further explored.

The *STK10* transcript was presented in several tissues and expressed highly in hematopoietic cells [34]. It had been found that *STK10* mutations were associated with hematological diseases, like peripheral T-cell lymphoma (PTCL) and Burkitt lymphoma (BL) [4, 33]. Our results confirmed *STK10* possessed the cell specificity in blood & immune cells and overexpressed especially in AML, maybe serving as a promising marker in predicting the malignant transformation to AML.

Several studies related to the *STK10* in cancers have defined the association of *STK10* with diverse outcomes depending on the type of cancer [2]. In the current study, we demonstrated that high *STK10* expression carried an increased risk of poor OS in AML. The same findings were also identified in the subgroups of the patients ≤ 60 years old or with the non-high-risk cytogenetics. Furthermore, the low expression of *STK10* might become an indication of distinguishing the patients with favorable cytogenetics, including inv(16), t(8;21) and t(15;17), from the unfavorable cytogenetic risks.

A slice of studies reported *STK10* was mainly expressed in spleen, thymus, BM, placenta and peripheral blood leukocytes, indicating the potential pathogenetic significance in hematopoietic tissues [3, 34]. Consistent with the above findings, our functional analysis manifested that *STK10* associated genes showed functional specificity mainly in blood, spleen and BM. We also identified these genes were associated with several biological processes of leukemia, like regulation of cytokine production, phagocytosis, myeloid leukocyte activation, leukocyte migration and tumor necrosis factor superfamily cytokine production, all of which were related to the origin, pathophysiology and histopathology of AML [35]. Besides, we reported two potential genes, which directly interacted with *STK10*, influenced the survival of AML patients. *ITGB2* and *ITGAM* have tissue specificity on BM [2] and take part in a portion of vital biological processes. For example, *ITGB2* is involved in adhesion, migration of leukocytes [36] and immune response including NK cell-induced cytotoxicity [37–39]. From a bioinformatics analysis, *ITGAM* and *ITGB2* were also up-regulated in myeloma and involved in cytokine-cytokine receptor interaction, innate immune response and inflammatory response that were similar to our results [40]. Moreover, the integrin ITGAM/ITGB2, associated with immunity and inflammation [41, 42], was gradually increased along with hematopoietic stem cells (HSC) differentiation in mice [41], suggesting the indirect role of STK10 in immune and defense. However, this view still needs to be verified by further studies.

It is universally acknowledged that immune dysregulation is a general feature in most of cancers including AML. Infiltrating immune cells participate in the regulation of quite a few biological processes, like chemotherapy, immunotherapy, immune escape and disease progression [43, 44]. Our work implicated that both CD56^dim^ NK cells and CD8 + T cells are positively associated with *STK10*. The mature CD56^dim^/NKG2A-/KIR+/CD57 + NK cells developing from CD56^bright^/NKG2A + NK cells can exert greater cytotoxicity to HLA-E + target cells [30, 45, 46]. Previous studies reported that NKG2A, as an inhibitory receptor, was overexpressed in AML and associated with failure to achieve remission [46]. Consistent with these results above, we found HLA-E, the ligand for NKG2A on cancer cells, was also overexpressed indicating poor OS in AML. Moreover, our work unraveled that *STK10* was positively correlated with the expression of *HLA-E*, potentially accounting for the crosstalk of *HLA-E* and *STK10*. Besides, NKG2A was also expressed on CD8 + T cells and identified as a new immune checkpoint [47, 48].

The rationale for widespread exploration of programmed cell death 1 protein (PD-1) inhibitors in AML is supported by the increased expression of programmed cell death ligand 1(PD-L1, CD274) and its prognostic value [49]. By blocking either the co-inhibitory receptor PD-1 or its ligand PD-L1, PD-1 inhibitors activate T cell-induced antitumor activity [32, 49]. T-cell immunoglobin mucin-3 (TIM-3) which could be activated by GAL-9 acted as an inhibitory receptor expressed on T cells [32]. Therefore, we studied ligands expression of these immune checkpoints. In our analysis, the expressions of *GAL-9* and *CD274* were higher in patients with AML, indicating poor prognosis as well. Moreover, a positive correlation between these immune checkpoints with *STK10* expression was also observed. All the findings above suggested an immunoregulatory effect of *STK10*.

In summary, we clarified that *STK10* was overexpressed in tumor cells and correlated with an unfavorable prognosis in AML. Functional studies revealed the close association of *STK10* with several biological processes in AML. Besides, we identified two potential genes that may tightly interact with *STK10* in the pathogenesis of AML, which required further investigation. Our findings also suggested that *STK10* might influence immune cell infiltration and was associated with immune escape by interacting with some immune checkpoints. Taken together, our work led to the first expansion of our understanding of the significance of *STK10* as a new prognostic factor or therapeutic target for AML.

## Supplementary Information

**Additional file 1.** Univariate and Multivariate analyses based on clinical characteristics and the expression of STK10.

**Additional file 2.** The differential expressed RNA between *STK10*^high^ and *STK10*^low^ groups identified by R and limma.

**Additional file 3.** The correlation of STK10 with other genes analyzed by the Spearman’s test.

**Additional file 4.** Network of the 172 proteins from the overlapping genes, available in String database version 11.0b.

**Additional file 5.** Verification of *ITGB2*, *ITGAM*, *HLA-E* and *LGALS9* in GSE9476.

**Additional file 6.** Univariate and Multivariate analyses based on the expression of genes associated with *STK10* directly.

## Data Availability

The datasets used and/or analysed during the current study are available from NCBI Gene Expression Omnibus (GEO: GSE9476) https://www.ncbi.nlm.nih.gov/geo/ and the cancer genome database (TCGA: LAML) https://portal.gdc.cancer.gov.

## Abbreviations

AML: Acute myeloid leukemia
BM: Bone marrow
*STK10*: *Serine/threonine kinase 10*
*LOK*: L*ymphocyte-oriented kinase*
MAPK: Mitogen-activated protein kinase
*PLK1*: *polo-like kinase 1*
ERM: Ezrin-radixin-moesin
*EZR*: *Ezrin*
HPA: Human protein atlas
TPM: Transcripts per million
GEPIA: Gene expression profiling interactive analysis
TCGA: The cancer genome atlas
GTEx: The genotype-tissue expression project
FPKM: Per kilobase per million
GEO: Gene expression omnibus
GO: Gene ontology
KEGG: Kyoto encyclopedia of genes and genomes
PPI: Protein–protein interactions
FAB: French–American–British
WBC: White blood cell
PB: Peripheral blood
Log_2_FC: Log2 fold change
OS: Overall survival
ROC: Receiver operating characteristic
AUC: Area under the curve
K-M: Kaplan–Meier
TReg: Regulatory T cells
TFH: T follicular helper
CTLs: Cytotoxic T lymphocytes
PTCL: Peripheral T-cell lymphoma
BL: Burkitt lymphoma
HSC: Hematopoietic stem cells
PD-1: Programmed cell death 1 protein
PD-L1: Programmed cell death ligand 1
TIM-3: T-cell immunoglobin mucin-3
