# Supplementary material for: Systematic analysis of prognostic significance, functional enrichment and immune implication of STK10 in acute myeloid leukemia
Source: BMC Med Genomics. 2022 May 1;15:101. doi: 10.1186/s12920-022-01251-7 (PMC9063138; doi:10.1186/s12920-022-01251-7)
Supplement: Supplementary file 1 — Additional file 1. Univariate and Multivariate analyses based on clinical characteristics and the expression of STK10. [file 12920_2022_1251_MOESM1_ESM.docx]

Additional file 1. Univariate and Multivariate analyses based on clinical characteristics and the expression of STK10

| Characteristics | Total(N) | Univariate analysis | | Multivariate analysis | |
| --- | --- | --- | --- | --- | --- |
|  |  | Hazard ratio (95% CI) | P value | Hazard ratio (95% CI) | P value |
| **Gender** | 140 |  |  |  |  |
| Female | 63 | Reference |  |  |  |
| Male | 77 | 1.030 (0.674-1.572) | 0.892 |  |  |
| **Race** | 137 |  |  |  |  |
| Black or African American | 10 | Reference |  |  |  |
| White | 127 | 1.383 (0.506-3.780) | 0.527 |  |  |
| **Age** | 140 |  |  |  |  |
| <=60 | 79 | Reference |  |  |  |
| >60 | 61 | 3.333 (2.164-5.134) | **<0.001** | 2.961 (1.871-4.684) | **<0.001** |
| **WBC count(x10^9/L)** | 139 |  |  |  |  |
| <=20 | 75 | Reference |  |  |  |
| >20 | 64 | 1.161 (0.760-1.772) | 0.490 |  |  |
| **BM blasts(%)** | 140 |  |  |  |  |
| <=20 | 59 | Reference |  |  |  |
| >20 | 81 | 1.165 (0.758-1.790) | 0.486 |  |  |
| **PB blasts(%)** | 140 |  |  |  |  |
| <=70 | 66 | Reference |  |  |  |
| >70 | 74 | 1.230 (0.806-1.878) | 0.338 |  |  |
| **Cytogenetic risk** | 138 |  |  |  |  |
| Favorable& Intermediate | 31 | Reference |  |  |  |
| Poor | 107 | 1.809 (1.105-2.961) | **0.018** | 1.286 (0.772-2.140) | 0.334 |
| **STK10*** | 140 |  |  |  |  |
| Low | 74 | Reference |  |  |  |
| High | 66 | 1.922 (1.254-2.945) | **0.003** | 1.562 (1.001-2.438) | **0.050** |

*: The expression of STK10 is grouped by the median value (77.64).
