# Supplementary figures and images for: Systematic analysis of prognostic significance, functional enrichment and immune implication of STK10 in acute myeloid leukemia
Source: BMC Med Genomics. 2022 May 1;15:101. doi: 10.1186/s12920-022-01251-7 (PMC9063138; doi:10.1186/s12920-022-01251-7)

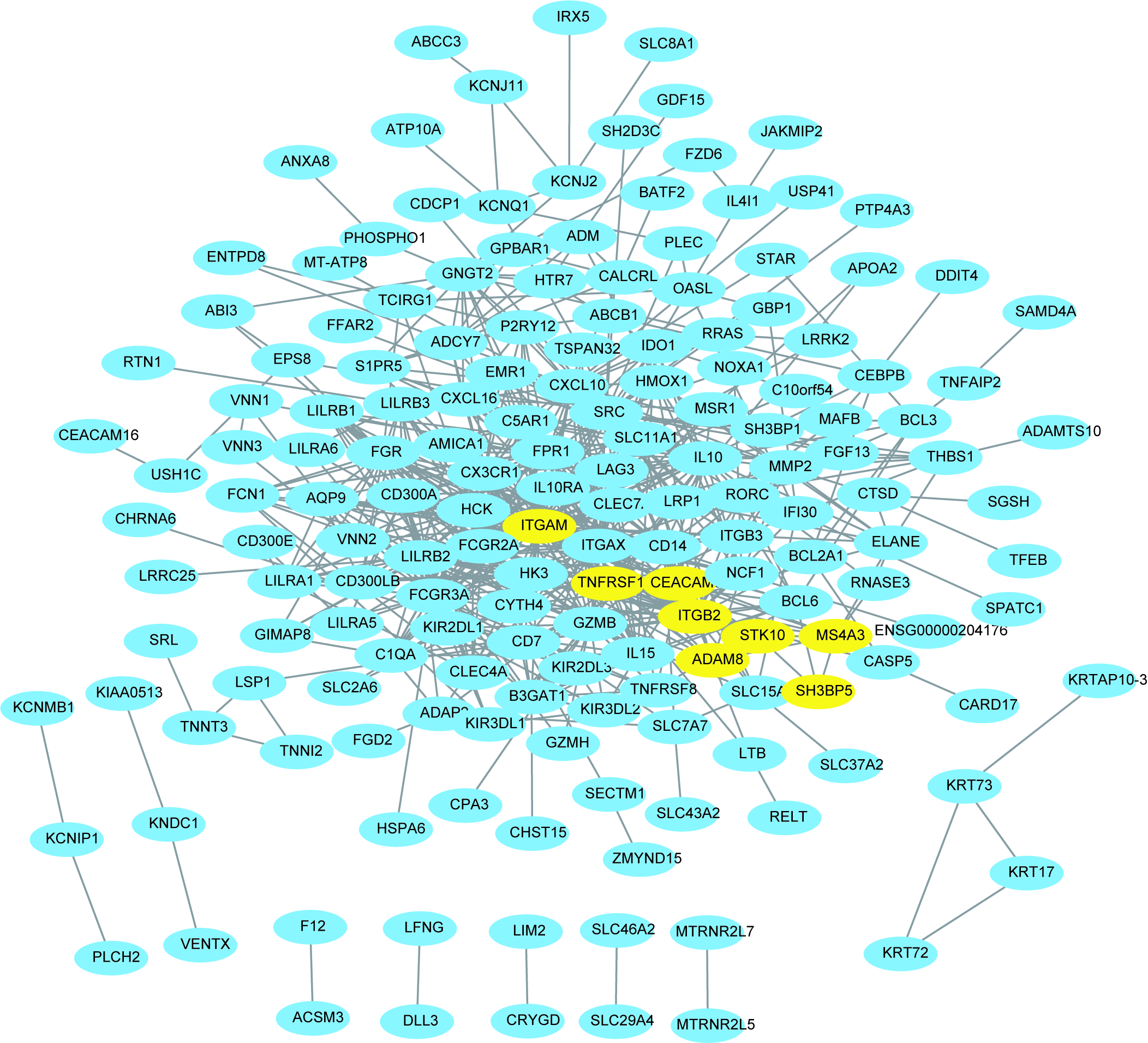

Supplement: Supplementary file 4 — Additional file 4. Network of the 172 proteins from the overlapping genes, available in String database version 11.0b. [file 12920_2022_1251_MOESM4_ESM.tif]

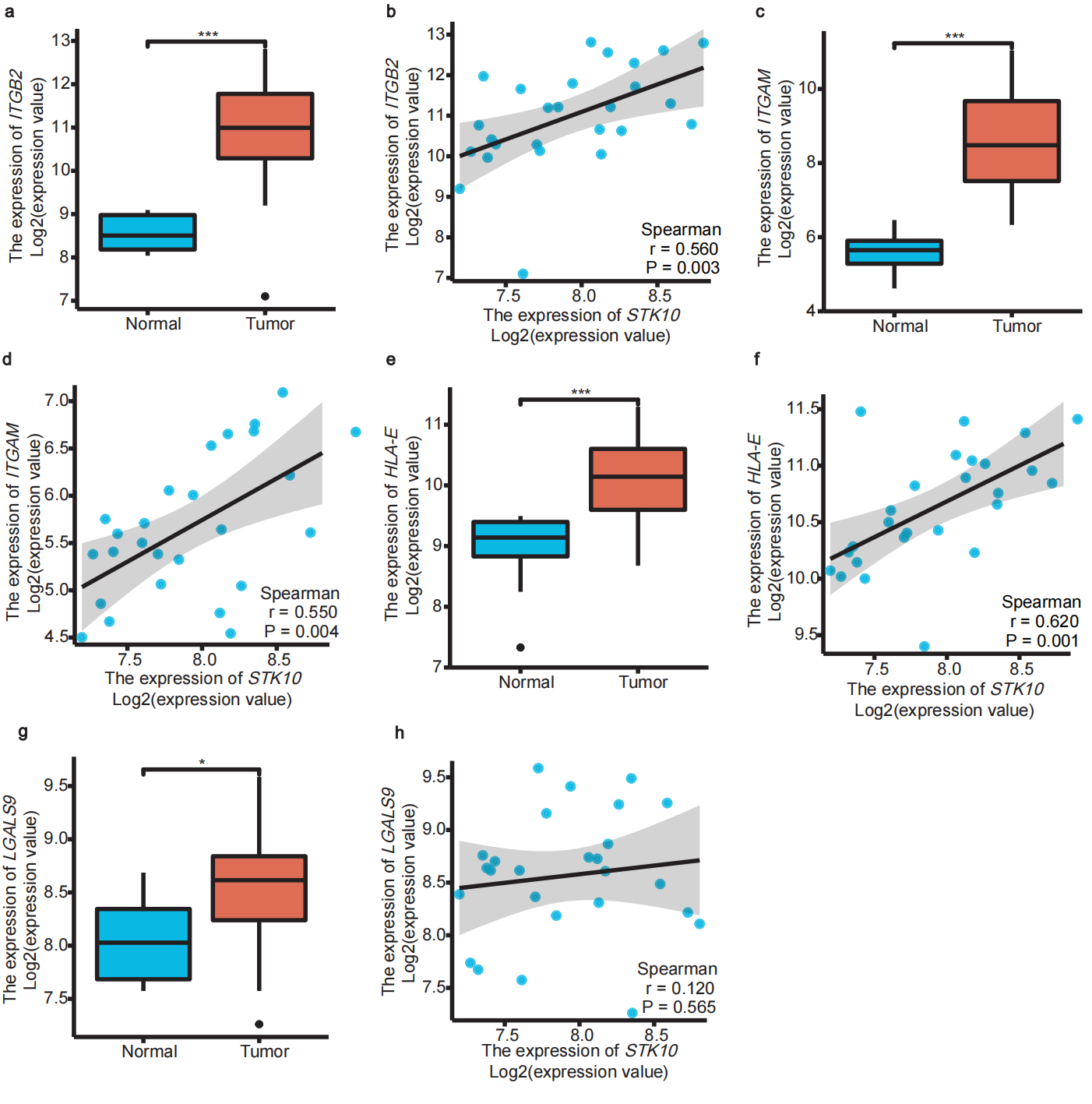

Supplement: Supplementary file 5 — Additional file 5. Verification of ITGB2, ITGAM, HLA-E and LGALS9 in GSE9476. [file 12920_2022_1251_MOESM5_ESM.tif]
