# Supplementary material for: Systematic analysis of prognostic significance, functional enrichment and immune implication of STK10 in acute myeloid leukemia
Source: BMC Med Genomics. 2022 May 1;15:101. doi: 10.1186/s12920-022-01251-7 (PMC9063138; doi:10.1186/s12920-022-01251-7)
Supplement: Supplementary file 6 — Additional file 6. Univariate and Multivariate analyses based on the expression of genes associated with STK10 directly. [file 12920_2022_1251_MOESM6_ESM.docx]

Additional file 6. Univariate and Multivariate analyses based on the expression of genes associated with STK10 directly.

| Characteristics | Total(N) | Univariate analysis | | Multivariate analysis | |
| --- | --- | --- | --- | --- | --- |
|  |  | Hazard ratio (95% CI) | P value | Hazard ratio (95% CI) | P value |
| **ITGAM** | 140 |  |  |  |  |
| Low | 71 | Reference |  |  |  |
| High | 69 | 1.616 (1.056-2.474) | **0.027** | 1.110 (0.636-1.936) | 0.713 |
| **ITGB2** | 140 |  |  |  |  |
| Low | 70 | Reference |  |  |  |
| High | 70 | 1.912 (1.242-2.943) | **0.003** | 1.490 (0.825-2.693) | 0.187 |
| **STK10** | 140 |  |  |  |  |
| Low | 74 | Reference |  |  |  |
| High | 66 | 1.922 (1.254-2.945) | **0.003** | 1.610 (1.015-2.554) | **0.043** |
| **CEACAM3** | 140 |  |  |  |  |
| Low | 71 | Reference |  |  |  |
| High | 69 | 1.105 (0.724-1.684) | 0.644 |  |  |
| **ADAM8** | 140 |  |  |  |  |
| Low | 69 | Reference |  |  |  |
| High | 71 | 1.431 (0.937-2.185) | 0.097 |  |  |
| **SH3BP5** | 140 |  |  |  |  |
| Low | 70 | Reference |  |  |  |
| High | 70 | 1.375 (0.901-2.097) | 0.140 |  |  |

Genes entered the univariate and multivariate analyses are directly associated with STK10 from protein-protein analysis (Figure 6a), and upregulated or downregulated in tumor samples from AML. Median value of genes’ expression was adopted for grouping.
